# Quick and Sensitive Detection of Water Using Galvanic-Coupled Arrays with a Submicron Gap for the Advanced Prediction of Dew Condensation

**DOI:** 10.3390/s20113314

**Published:** 2020-06-10

**Authors:** Rekha Goswami Shrestha, Yusuke Kubota, Yukihiro Sakamoto, Jin Kawakita

**Affiliations:** 1 Electric and Electronic Materials Field, Electrochemical Sensors Group, Research Center for Functional Materials, National Institute for Materials Science, Ibaraki 305-0044, Japan; rekhashrestha3@hotmail.com (R.G.S.); KUBOTA.Yusuke@nims.go.jp (Y.K.); 2 Graduate School of Engineering, Chiba Institute of Technology, Chiba 275-0016, Japan; yukihiro.sakamoto@it-chiba.ac.jp

**Keywords:** galvanic current, electrodes, thin film of water molecules, cooling rate, vapor pressure, sensitivity and accuracy

## Abstract

We have demonstrated a highly sensitive moisture sensor that can detect water molecules, in addition to water droplets, and therefore, can predict dew condensation with high accuracy and high speed before the formation of water droplets, showing a better performance than a commercial hygrometer. Additionally, the dependence of the output response from the sensor on factors, such as the cooling rate of the sensor’s surface and the vapor pressure in the chamber, that affect the performance of the moisture sensor has been clarified. The output response showed a clear dependence on the variation in cooling rate, as well as the vapor pressure. The higher the cooling rate and vapor pressure, the higher the output response. The output response showed a linear response to the change in the above-mentioned parameters. The higher sensitivity and accuracy of the moisture sensor, as a function of the physical parameters, such as cooling rates, vapor pressure, enables the sensor to perform in advanced detection applications. The sensor can be modified to the actual target regarding the surface nature and the heat capacity of the target object, making it more suitable for wide applications.

## 1. Introduction

There are numerous applications where sensors are widely used, such as air conditioners and humidifiers, industrial process control, medical science, food production, agriculture, weather bureaus, meteorological, and marine monitoring [1,2,3,4,5,6,7,8,9,10,11,12]. They require precisely controlled humidity levels and rely on accurate and fast humidity sensing. Electronic humidity sensors are cheaper, lighter, and smaller compared to existing infrared humidity sensors, which makes them more suitable for sensor networks. Portable, reliable, and low-cost humidity sensors play an important role in our day-to-day life, including in industry, agriculture, environmental fields [13], and medical devices [2]. Nonetheless, high-precision, accurate, fast-response humidity sensors are important [14]. Accuracy and precision are the most important characteristics of a humidity sensor. Several problems can occur while using humidity sensors for humidity detection, such as low sensitivity, delayed response and recovery times, less stability, and narrow humidity detection ranges.

Commercial hygrometers, such as polymer condensation sensors [2], cannot detect actual dew condensation because their working principle is based on absorbing water molecules of vapor in the atmosphere with hygroscopic material. They cannot distinguish between vapor and liquid phases of water. They need some time to reach stable values and beyond 80% relative humidity, the reliability of measurement is decreased. The water droplet size plays a crucial role in the measurement and control of condensation, and some cannot distinguish their shape and size. A few commercial techniques can determine the diameter of water droplets, but only those droplets larger than 10 μm. The chilled mirror dew point hygrometers [2], whose detection principle is based on changes in reflection due to the formation of dew, which is not a direct and precise method for detection. Furthermore, the apparatus used for measurement are often large and multiple devices have to be fixed for their functional operation. There exist no commercial technologies for detecting small water molecules yet (≈0.5 μm or less). Additionally, to maintain an optimal environment, it is essential to have a suitable humidity sensor that can precisely detect and control the ambient environment under different conditions, including an abrupt change of temperature [15]. A humidity sensor optimized for measurement with high accuracy and precision is highly desired [16]. Table 1 shows the comparison of various commercial sensors used for measuring humidity and dew condensation.

In this context, NIMS (National Institute for Materials Science) has developed a small, portable, reliable moisture sensor that can detect and measure the diameter of a microscale/sub-microscale water droplet by measuring the current that flows spontaneously due to galvanic action with high sensitivity and high accuracy [17]. It measures the galvanic current generated across the electrodes, where a drop of water establishes a bridge-like structure. The electrodes are thin wires (arrays) of dissimilar metals arranged in gaps. By using a semiconductor fabrication process, the gap can be aligned to a relatively fine scale that is micro/submicron in size, leading to high detection sensitivity. The sensor can show an instantaneous signal upon the introduction of water molecules/droplets (within 20 milliseconds) on the sensor surface and turns off on complete evaporation, leading to a quick response. With a lower spacing between the electrodes, such as 0.5 μm, the sensor can even detect much smaller water molecules [18]. Further, the sensor’s surface [19] and the sensor as a whole can be modified to the actual target [20]. By using these features, this sensor is expected to detect dew condensation with high accuracy at an early stage and is catered to the actual target. We have demonstrated the detection of micro/nano-sized water droplets with a quick response of 200 ms [18] and the improvement of its sensitivity and accuracy via the modification in the surface of the sensor chip [19]. Additionally, we suggested the prediction of dew condensation from sensing behavior at different but steady humidity around 100%. So far, the temperature of this sensor was kept constant in almost all cases when its sensing behavior was investigated. Since the current response from this sensor depends on the state (shape and size) of water molecules/droplets present on the sensor’s surface, it is necessary to understand and establish the relationship between the water droplet’s state and its corresponding current response.

In this study, we demonstrated a highly sensitive moisture sensor that can detect water molecules, in addition to water droplets, and therefore, can predict dew condensation before the formation of water droplets. It is very important to clarify the effects of cooling rate on dew condensation as targets can have different responses to the cooling rates depending upon their heat capacity, and ultimately the dew condensation. Here, we have studied the relationship between the output response from the sensor and factors, such as cooling rate of the sensor’s surface and vapor pressure in the chamber, that affect the performance of the moisture sensor, and clarified the effect of the cooling rate of sensor’s surface on the current response of the moisture sensor corresponding to the stacking of adsorbed water molecules. We have demonstrated the performance, sensitivity, and accuracy of the sensor when measuring small water molecules. We presume that the present research can address the demands for a precise and accurate moisture sensor and stimulate new experimental research in terms of better sensitivity and low response time and try to explore its potential for future humidity-sensing applications.

## 2. Materials and Methods

### 2.1. Preparation of the Sensor Chip

**Figure 1:**
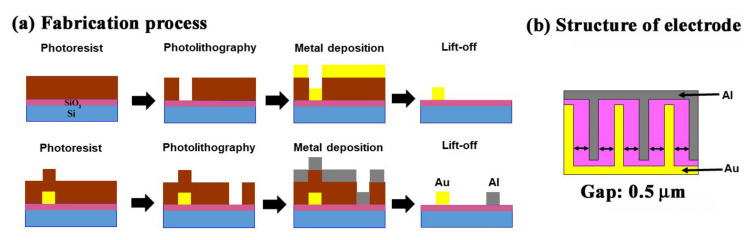
(**a**) Schematic flow of the fabrication process of a sensor with the arrangement of Al and Au electrodes on the SiO_2_ substrate, and (**b**) the structure and placements of electrodes.

The sensor chip was made using a semiconductor microfabrication technique [17], as demonstrated in Figure 1a. The sensor had a two comb-shaped structure made of two different metals intercalating with each other with a narrow gap in between each electrode. These comb-like structures were placed on the surface of SiO_2_-coated Si wafer (3-inch Si(100) wafer with thermally oxidized SiO_2_ (200 nm) layer and conductivity p-type, 1–10 ohm cm) with a 4-inch diameter used as the substrate. The coating of the Si wafer with an insulating layer of silicon oxide was performed via thermal oxidation. Photolithography and metal deposition processes were implemented to fabricate the inter-digit arrays made of gold (Au) (purity: “4Nup”) and aluminum (Al) (purity: “4N”) metals facing each other on a silica layer, as shown in Figure 1. Then, chips approximately 5 mm in size were cut out of the processed wafer. A schematic illustration of the arrays’ placement is shown in Figure 1b. The electrode gap was set at 0.5 μm. The width and the thickness of electrodes were 1 μm and 150 nm, respectively, and the number of electrode pairs was 50.

### 2.2. Introduction of Droplets on the Sensor’s Surface

**Figure 2:**
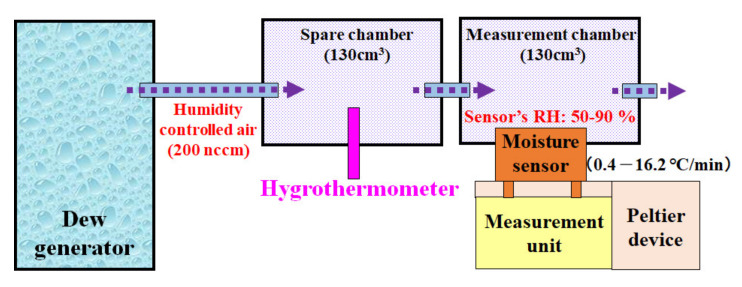
Schematic diagram showing the experimental setup for the introduction/adsorption of water droplets onto a moisture sensor by introducing humidity-controlled air while measuring the current. The sensor was cooled using a Peltier device from 35 °C to 0 °C. For a precise generation of the defined relative humidity at a defined air temperature, a humidity generator was used. The preset humid air was then passed in with a flow rate of 200 nccm.

Figure 2 shows the overall scheme of the experimental setup. To conduct the measurement, the precise control of humidity and temperature was required; therefore, we used a custom-made setup. A measurement device with a moisture sensor was placed in a measurement chamber. The relative humidity of the chamber was controlled by a precise humidity control generator (Micro Equipment Inc., me-40DPRT-2FM-MFC, Tokyo, Japan). Then, the measurement chamber was connected to another chamber with the same volume in series on one side with a hygrometer attached. Using this set up, we measured the temperature and humidity precisely using a capacitive hygrometer (E + E Elektronic/EE23, Austria). Humidity-controlled air at 50–90% relative humidity (RH) with a flow rate of 200 nccm was introduced into the chamber. Then, dew condensation was induced on the sensor surface by cooling the backside of the sensor using a Peltier device (Ampere Co., Ltd., Tokyo, Japan), which cooled the surface from 35 °C at different cooling rates (0.4 to 16.2 °C/min). The temperature of the sensor was estimated using the Pt wires on the sensor chip from the correlation line of its temperature and electrical resistance, in advance of the actual experiment.

### 2.3. Measurement of the Sensor Output

**Figure 3:**
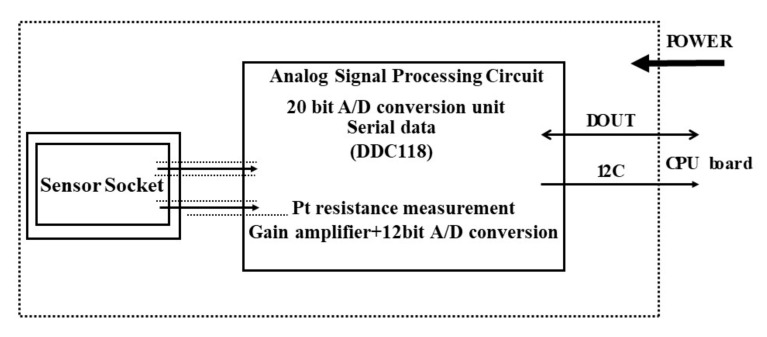
Schematic diagram showing the measurement of the sensor output.

The sensor chip was attached to a custom-made measurement unit (Figure 2 and Figure 3). The current was measured using a hand-made device that included a 20-bit, octal channel, current-input analog-to-digital (A/D) converter (DDC118, Texas Instruments, Tokyo, Japan) that could measure currents in the fA to µA range. The data was then collected as time–current data using the software on the PC (the platform is Processing and we have made program by ourselves according to our need). The output of this device was calibrated using a semiconductor evaluation instrument (Agilent Technologies B1500A, Agilent Technologies, Tokyo, Japan).

## 3. Results and Discussion

**Figure 4:**
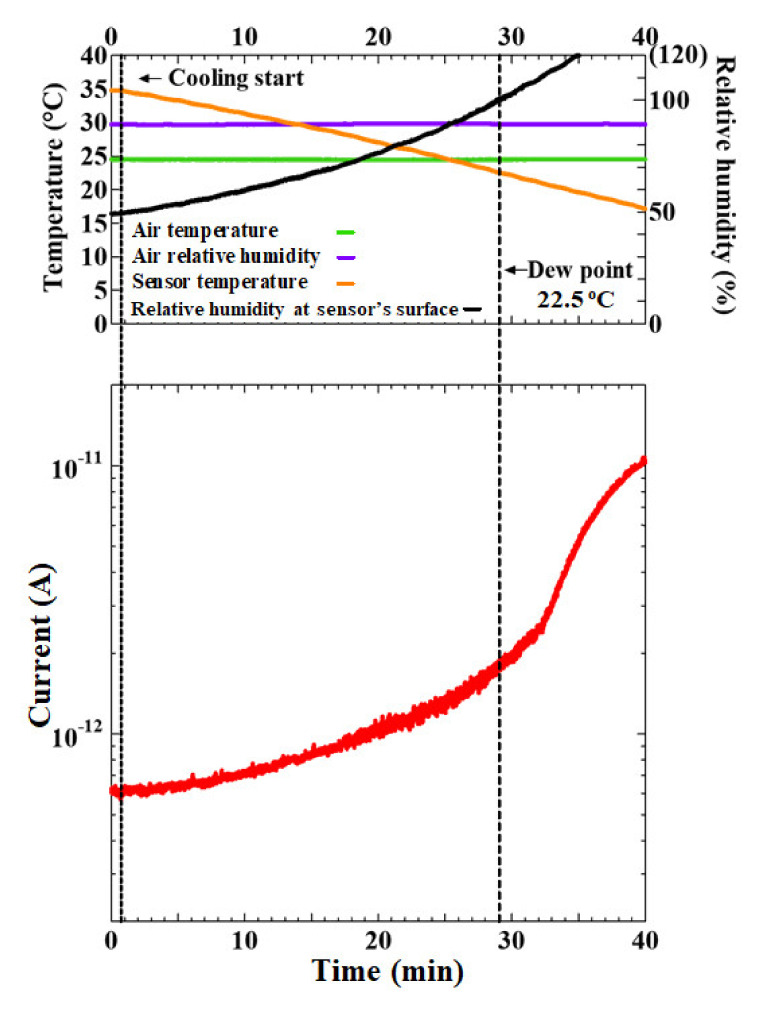
Output current over time (red line) as the sensor’s surface was cooled from 35 °C to ≈ 0 °C at 0.5 °C/min, at 1.54 kPa vapor pressure, and with a 90% relative humidity inside the measurement chamber.

Figure 4 shows the output current when the temperature of the sensor’s surface was cooled down from 35 °C at 0.5 °C/min rate, at 1.54 kPa vapor pressure, and with a 90% relative humidity inside the measurement chamber.

Figure 4 shows that the current immediately started increasing gradually when the sensor’s surface was cooled at a rate of 0.5 °C /min under a humidity-controlled environment. The sensor showed a steadily increasing output response, even before the dew condensation started (dew point = 22.5 °C). The water molecules presumably started to appear on the cooled sensor surface and formed thin films bridging the two dissimilar electrodes to generate a galvanic current [18]. The moisture sensor was very fast at showing an output response in comparison to a hygrometer. The calculated relative humidity near the sensor’s surface (black line) reached 100% at a point (dew point = 22.5 °C); beyond this point, the response further increased abruptly due to the formation of water droplets [19]. From this observation, it can be said that it was possible to detect minute and relatively gradual changes in humidity with high accuracy and high speed. Therefore, the moisture sensor could precisely detect the dew condensation in advance such that necessary preventive measures can be realized, unlike with a commercial hygrometer.

### Dependence of the Sensor’s Output on Vapor Pressure and Cooling Rates of the Sensor’s Surface

**Figure 5:**
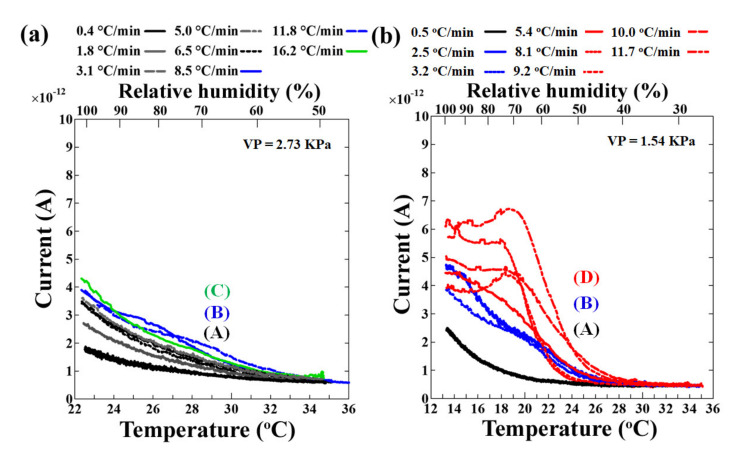
Output response (current) as a function of the temperature of the sensor’s surface being cooled at different cooling rates from 35 °C at (**a**) 2.73 kPa and (**b**) 1.54 kPa vapor pressure (VP). The output has been shown till 100% relative humidity in each case. The types of current increasing trends observed at 2.73 kPa and 1.54 kPa vapor pressure have been assigned (**A**) for black lines, (**B**) for blue lines, (**C**) for green line, and (**D**) for red lines.

The sensor’s surface was cooled from 35 °C at different rates and the output currents were observed at 2.73 kPa and 1.54 kPa vapor pressures inside the chamber. Figure 5 shows the overall results of the variation of output current with a change in humidity near the sensor’s surface and the cooling rates of the sensor’s surface.

When the surface of the sensor was cooled, a rise in current from the background level was observed at all cooling rates and vapor pressures studied (Figure 5a,b). It was found that the slower the cooling rates, the slower the increase in current, and the higher the cooling rates, the higher the output current, while keeping other parameters constant. At a 2.73 kPa vapor pressure, three types of increasing trends were observed: (A) black lines, (B) blue lines, and (C) green line (Figure 5a). Similar current trends were observed at a 1.54 kPa vapor pressure too: (A) black lines and (B) blue lines (Figure 5b). However, as the cooling rate was further increased (5.4 °C/min and beyond), a slightly different trend was observed, namely the (D) red lines in Figure 5b, where the output current became almost constant and later decreased.

The slope remained linear relative to the change in temperature and humidity, which means it was very sensitive toward the change in temperature and humidity, where the output response could be detected with high speed and high accuracy from the moment the cooling starts. In conclusion, the sensor instantaneously generated a current response due to the adhesion of minute water molecules/droplets in between the electrodes, and the current depended on the coverage status of the sensor surface, i.e., bridging the space between the electrodes. These results showed that the output response was dependent on the cooling rate of the sensor’s surface, as well as the vapor pressure of the chamber.

**Figure 6:**
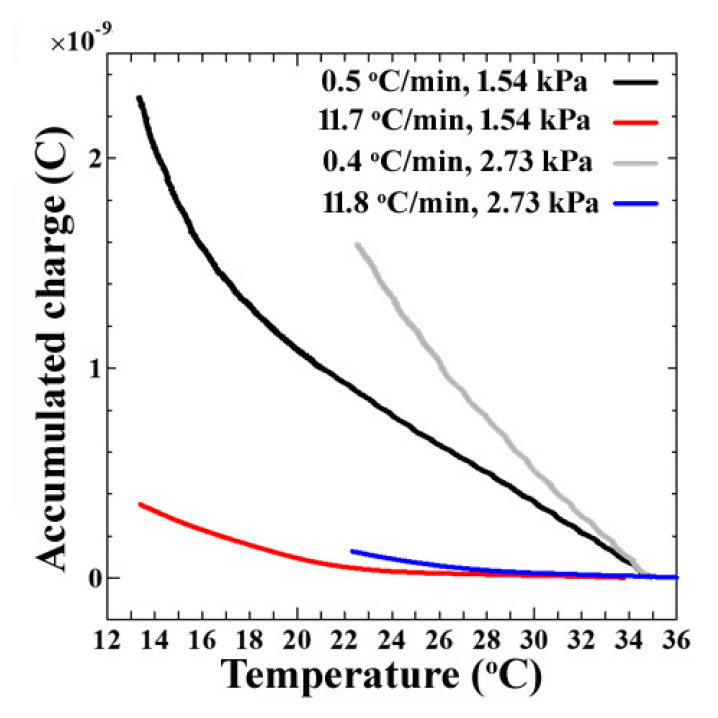
The accumulated charge as a function of the temperature of the sensor’s surface being cooled at 0.5 °C/min or 11.8 °C/min rates from 35 °C at 2.73 kPa or 1.54 kPa vapor pressure.

To explain the reasons behind the variations in the output from the sensor as a function of cooling rates and the vapor pressure, the corresponding accumulated charge was calculated as a function of the temperature of the sensor’s surface at cooling rates of 0.5 °C/min and 11.8 °C/min at both 2.73 kPa and 1.54 kPa vapor pressure. Figure 6 shows the overall results.

**Figure 7:**
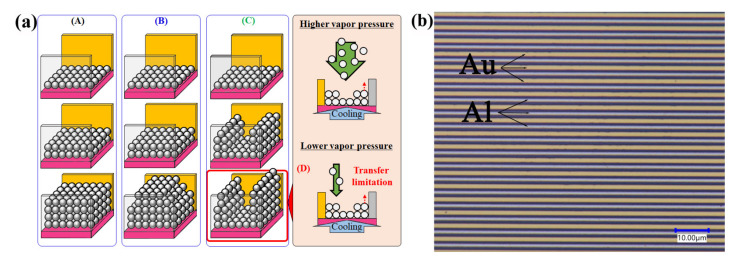
(**a**) Scheme showing the probable reasons for the difference in output current as a function of cooling rates at different vapor pressures and (**b**) an optical micrograph of the sensor’s surface with a 0.5 mm gap between the electrodes at 70% RH near the sensor’s surface.

The plots of the accumulated charge as a function of the temperature of the sensor’s surface show that for both vapor pressures, as the cooling started, there was an accumulation of charge along the surface, increasing the current response. When we closely observe the trend in Figure 6, at 2.73 kPa (gray and blue) and 1.54 kPa (black and red), at almost the same cooling rate (black and gray) and (blue and red), it was found that the accumulated charge was comparatively higher in the case of the high vapor pressure, corresponding to the same temperature of the sensor’s surface. It was due to the availability of a higher number of water molecules suspended in the humidity-controlled air in the chamber at a higher vapor pressure. Here, the adsorption of water molecules, but not water droplets, was expected since the liquid water droplet was not visible on the sensor’s surface between the electrodes in the optical micrographs, yet an increase in the output current was observed before 100% RH (Figure 7b). It has been shown through other techniques, such as QCM (Quartz crystal microbalance), that the theory of the adsorption of water molecules with an increase in relative humidity in the chamber before dew-condensation occurs before 100% RH is correct [21,22]. Therefore, the probable mechanism for the difference in the output current as a function of cooling rates at different vapor pressures can be visualized as in Figure 7a.

At both 1.54 kPa and 2.73 kPa vapor pressures, when cooling the sensor’s surface at lower cooling rates, the output current trend suggested that at first, the water molecules were adsorbed in between the electrodes and were further stacked vertically and horizontally in layers, increasing the current (Figure 5(A) and Figure 7(A)). With increasing cooling rates, water molecules preferred vertical stacking in comparison to horizontal stacking such that minimal horizontal stacking occurred (Figure 5(B) and Figure 7(B)). With further increases in the cooling rates, especially at the lower vapor pressure, the preference of water molecules for vertical stacking over horizontal stacking was more than in (B). Then, the current became almost constant and a slight decrease was observed with a further increase in the cooling rates (red lines in Figure 5b and Figure 7a(D)). The low vapor pressure meant fewer water molecules were available in the humidity-controlled air within the chamber, and hence, there was a limitation in the exchange of water molecules in between the vapor state and the adsorbed water molecules.

**Figure 8:**
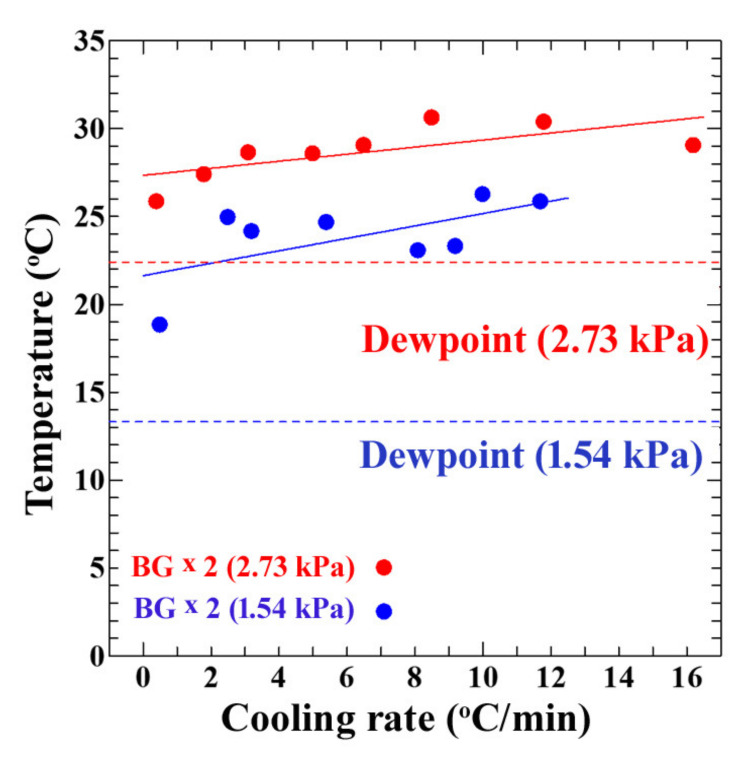
The temperature at which the sensor reached twice the background current as a function of the cooling rates of the sensor’s surface at 2.73 kPa (red data points) and 1.54 kPa (blue data points) vapor pressures.

To compare the sensor’s sensitivity and accuracy, the temperature at which its output current reached twice its background current (to avoid noise) under controlled humid conditions was plotted against the cooling rates of the sensor’s surface at both vapor pressures (1.54 kPa and 2.73 kPa). Figure 8 shows the overall results.

It was found that the sensor showed an instantaneous output response and the temperature showed a linear relationship with the increase in cooling rate, even before the dew point. This means that it could detect water molecules well before the adsorption of water droplets, which is dew condensation. This allowed for the advanced detection of dew condensation and steps could be undertaken to avoid the adverse effects of dew condensation. From these results, it is suggested that the moisture sensor can be used for early dew detection through a wide range of environments with high accuracy.

Therefore, this research has shown that the moisture sensor showed a clear sensitivity and quick response regarding the detection of invisibly small water molecules. The output response (sensing response) from the sensor was due to the galvanic effects from the introduction of a liquid film of water molecules that bridged between the two dissimilar electrodes. The moisture sensor was very quick in responding to the water molecules attached to the sensor surface at different vapor pressures through a wide range of cooling rates, even before the actual dew condensation occurred. The output response was very accurate compared to the ambiguous response from the commercial hygrometer. We could modify and adjust the sensor, temperature, and humidity conditions to target actual droplet formation and control the sensing response.

## 4. Conclusions

The main conclusions of this study are as follows:

1. The moisture sensor showed high sensitivity and accuracy toward the detection of adsorbed water molecules in addition to water droplets.
2. Even in a phase preceding dew condensation, the adsorption of water molecules could be detected.
3. The relationship between the output response with variations of the cooling rate, initial temperature of the sensor’s surface, and water vapor pressure was clarified. The higher the cooling rate and vapor pressure, the higher the output response from the sensor.
4. The response of the moisture sensor to the variation in cooling rates allowed for the prediction of prior dew condensation, which depends upon the heat capacity of the targets.
5. The experimental parameters and conditions were optimized for the advanced dew/humidity detection.

This research has successfully reported a quick and accurate sensor response toward an attachment of invisibly small water molecules. The response was dependent on several parameters, such as cooling rate and vapor pressure, and was fast enough to detect water molecules before dew condensation occurred. Therefore, the desired degree of sensitivity and accuracy toward the detection of water molecules can be acquired by setting the parameters, and performance that is far better than the commercial hygrometer can be achieved. Therefore, this moisture sensor can find applications in the food and automobile industry, and several other applications, that rely on accurate and fast humidity sensing.

## Figures and Tables

**Table 1:** Comparison of various commercial sensors used for measuring humidity and dew condensation with a moisture sensor.

	Hygrometer	Dew Sensor	Dew Point Meter	Moisture Sensor
Detection of droplet (dew)	Not possible	Not possible	Possible	Possible (>0.2 μm)
Response time	10 s	10 s	about a min	<0.02 s
Control of sensor’s material	Limited control	Not possible	Not possible	Possible
Size	Palm	Palm	Desktop	Palm
